# Supplementary material for: Factors Associated with Colorectal Cancer Screening in Spain: Results of the 2017 National Health Survey
Source: Int J Environ Res Public Health. 2022 Apr 29;19(9):5460. doi: 10.3390/ijerph19095460 (PMC9100170; doi:10.3390/ijerph19095460)
Supplement: Supplementary file 1 [file ijerph-19-05460-s001.zip › ijerph-1689084-supplementary.pdf]

International Journal of
Environmental Research and Public Health

MDPI

HIGHLIGHTS

1. The coverage of CRC screening in Spain in patients aged between 50 and 69 is below the 2016 PAPPS recommendations.
2. The prevalence of participation in CRC screening in Spain in 2017 was 29%.
3. The coverage of CRC screening was varying among the different autonomous communities.
4. Having been born outside of Spain and not having been vaccinated against flu were factors associated with not having had a FOBT.
5. Participation in CCR screening increases with age.

Table S1. Absolute and relative frequencies of respondents who had/had not had a faecal occult blood test (FOBT) according to all explanatory variables.

	Total (N = 7568)		No FOBT (N = 5377, 71.0%)		Yes FOBT (N = 2191, 29.0%)		
**Explanatory variable**	**n**	**%**	**n**	**%**	**n**	**%**	***p* Value**
Autonomous community							
Basque Country	387	5.10%	107	27.70%	280	72.30%	<0.001
Navarre	108	1.40%	43	39.50%	65	60.50%	
Castile-León	436	5.80%	222	50.90%	214	49.10%	
Valencian Community	825	10.90%	454	55.10%	370	44.90%	
La Rioja	54	0.70%	31	56.80%	23	43.20%	
Cantabria	99	1.30%	60	60.30%	39	39.70%	
Canary Islands	352	4.60%	223	63.30%	129	36.70%	
Aragon	210	2.80%	146	69.50%	64	30.50%	
Murcia	219	2.90%	156	71.50%	62	28.50%	
Catalonia	1224	16.20%	905	73.90%	319	26.10%	
Balearic Islands	175	2.30%	130	74.60%	44	25.40%	
Galicia	454	6.00%	348	76.60%	106	23.40%	
Castile-La Mancha	336	4.40%	271	80.80%	65	19.20%	
Madrid	956	12.60%	781	81.70%	175	18.30%	
Asturias	208	2.80%	179	85.80%	30	14.20%	
Andalusia	1313	17.40%	1127	85.90%	186	14.10%	
Ceuta-Melilla	24	0.30%	22	89.60%	3	10.40%	
Extremadura	188	2.50%	172	91.30%	16	8.70%	
Age							
50-54 years	2367	31.3%	1841	77.8%	526	22.2%	<0.001
55-59 years	2047	27.0%	1441	70.4%	605	29.6%	
60-64 years	1666	22.0%	1116	67.0%	550	33.0%	
65-69 years	1488	19.7%	979	65.7%	510	34.3%	
Sex							
Man	3710	49.0%	2608	70.3%	1102	29.7%	0.157
Woman	3858	51.0%	2769	71.8%	1089	28.2%	
Body mass index							
Underweight/normal	2575	34.0%	1832	71.1%	743	28.9%	0.001
Overweight	3214	42.5%	2265	70.5%	949	29.5%	
Obesity	1582	20.9%	1115	70.5%	467	29.5%	
Don’t know/no answer	197	2.6%	165	83.6%	32	16.4%	
Country of birth							
Spain	6843	90.4%	4786	69.9%	2057	30.1%	<0.001

Outside Spain	725	9.6%	591	81.5%	134	18.5%	
Marital status							
Single	690	9.1%	509	73.8%	181	26.2%	0.164
Married	5883	77.7%	4157	70.7%	1726	29.3%	
Widowed	404	5.3%	292	72.3%	112	27.7%	
Separated	182	2.4%	139	76.0%	44	24.0%	
Divorced	408	5.4%	280	68.6%	128	31.4%	
Educational attainment							
Illiterate	88	1.2%	72	81.8%	16	18.2%	<0.001
Incomplete primary education	564	7.5%	436	77.4%	127	22.6%	
Primary education	1674	22.1%	1200	71.7%	474	28.3%	
Compulsory secondary education	1951	25.8%	1406	72.1%	545	27.9%	
Upper secondary education	988	13.1%	696	70.4%	292	29.6%	
Intermediate vocational training	526	7.0%	345	65.6%	181	34.4%	
Higher vocational training	461	6.1%	324	70.3%	137	29.7%	
University education	1317	17.4%	899	68.2%	419	31.8%	
Socio-occupational class							
Manager with ≥ 10 employees	813	10.7%	547	67.3%	266	32.7%	<0.001
Manager with < 10 employees	609	8.0%	439	72.2%	169	27.8%	
Intermediate occupations and own-account workers	1491	19.7%	1032	69.2%	459	30.8%	
Skilled technical occupations	1151	15.2%	795	69.1%	355	30.9%	
Skilled primary sector workers	2384	31.5%	1715	71.9%	669	28.1%	
Unskilled worker	1010	13.4%	758	75.0%	252	25.0%	
Don't know/no answer	110	1.5%	90	81.2%	21	18.8%	
Smoking							
Daily smoker	1805	23.8%	1372	76.0%	433	24.0%	<0.001
Nondaily smoker	164	2.2%	125	76.0%	39	24.0%	
Ex-smoker	2611	34.5%	1760	67.4%	851	32.6%	
Non-smoker	2987	39.5%	2120	71.0%	868	29.0%	
Alcohol consumption							
Daily	1822	24.1%	1283	70.4%	539	29.6%	0.061
5-6 days/week	134	1.8%	85	63.6%	49	36.4%	
3-4 days/week	308	4.1%	210	68.2%	98	31.8%	
1-2 days/week	1143	15.1%	809	70.8%	333	29.2%	
2-3 days/month	715	9.4%	505	70.6%	210	29.4%	
Once a month	392	5.2%	274	70.0%	117	30.0%	
Less than once a month	754	10.0%	566	75.1%	188	24.9%	
Nothing in the last 12 months	1088	14.4%	758	69.7%	330	30.3%	
Never	1213	16.0%	886	73.1%	327	26.9%	
Fruit and vegetable consumption							
No fruit or vegetables every day	1404	18.6%	1079	76.9%	325	23.1%	<0.001
Fruit or vegetables every day	3058	40.4%	2268	74.2%	790	25.8%	
Fruit and vegetables every day	3105	41.0%	2030	65.4%	1076	34.6%	
Main daily activity							
Mostly sitting	2397	31.7%	1643	68.5%	754	31.5%	0.016
Mostly standing	3661	48.4%	2638	72.0%	1024	28.0%	
Walking with some weight	1117	14.8%	804	72.0%	312	28.0%	
Manual labour	185	2.5%	141	76.2%	44	23.8%	
Not applicable	208	2.7%	151	72.7%	57	27.3%	
Leisure physical activity							

None	2713	35.8%	2002	73.8%	711	26.2%	<0.001
Occasional	3394	44.9%	2360	69.5%	1035	30.5%	
Several time a month	746	9.9%	487	65.3%	259	34.7%	
Several times a week	715	9.4%	528	73.9%	187	26.1%	
Tooth brushing							
Never	146	1.9%	118	80.5%	29	19.5%	0.002
Occasional	311	4.1%	223	71.5%	89	28.5%	
Once daily	1637	21.6%	1144	69.9%	493	30.1%	
Twice daily	3264	43.1%	2374	72.7%	890	27.3%	
Three or more times daily	2209	29.2%	1518	68.7%	691	31.3%	
Self-perceived health							
Very good	954	12.6%	721	75.5%	234	24.5%	<0.001
Good	3896	51.5%	2826	72.5%	1069	27.5%	
Fair	1948	25.7%	1329	68.2%	618	31.8%	
Poor	594	7.8%	380	64.0%	214	36.0%	
Very poor	176	2.3%	121	68.6%	55	31.4%	
Flu vaccine (last campaign)							
No	6150	81.3%	4495	73.1%	1655	26.9%	<0.001
Yes	1418	18.7%	882	62.2%	536	37.8%	
Colonoscopy (ever)							
No	6007	79.4%	4626	77.0%	1381	23.0%	<0.001
Yes	1561	20.6%	751	48.1%	810	51.9%	
Diabetes mellitus							
No	6757	89.3%	4856	71.9%	1901	28.1%	<0.001
Yes	811	10.7%	521	64.2%	290	35.8%	
High blood pressure							
No	5135	67.9%	3719	72.4%	1416	27.6%	<0.001
Yes	2433	32.1%	1658	68.2%	775	31.8%	
Blood/urine test*							
No	1592	21.0%	1279	80.3%	314	19.7%	<0.001
Yes	5976	79.0%	4098	68.6%	1877	31.4%	
X-ray*							
No	5359	70.8%	3873	72.3%	1486	27.7%	<0.001
Yes	2209	29.2%	1504	68.1%	705	31.9%	
Computed tomography scan*							
No	6715	88.7%	4843	72.1%	1872	27.9%	<0.001
Yes	853	11.3%	534	62.6%	319	37.4%	
Ultrasound*							
No	6207	82.0%	4504	72.6%	1704	27.4%	<0.001
Yes	1361	18.0%	873	64.2%	487	35.8%	
Magnetic resonance imaging*							
No	6709	88.6%	4821	71.9%	1888	28.1%	<0.001
Yes	859	11.4%	556	64.7%	303	35.3%	
Hospital admission*							
No	6895	91.1%	4935	71.6%	1961	28.4%	0.002
Yes	673	8.9%	442	65.8%	230	34.2%	
Emergency department visit*							
No	5599	74.0%	4022	71.8%	1577	28.2%	0.011
Yes	1969	26.0%	1355	68.8%	614	31.2%	
Primary care visit (last month)							
No	5034	66.5%	3705	73.6%	1329	26.4%	<0.001

Yes	2534	33.5%	1672	66.0%	862	34.0%

* in the last year.
